# Uncommon using of the pulmonary homograft in oncological case - three years follow up

**DOI:** 10.1186/s13019-024-02684-0

**Published:** 2024-04-13

**Authors:** Dhaker Lahidheb, Roman Komarov, Ruslan Alikhanov, Boris Tlisov, Alisher Ismailbaev, Ines Dhif

**Affiliations:** 1 Faculty of medicine of Tunis, University of Tunis El Manar, Tunis, Tunisia; 2 Department of Cardiac surgery, The First Clinical Hospital, Sechenov First Moscow State Medical University, Moscow, Russian Federation

**Keywords:** Pulmonary homograft, Right atrium thrombosis, Inferior vena cava thrombosis, Cardiopulmonary bypass, Surgical treatment

## Abstract

**Introduction:**

There are enough cases of colorectal cancer with liver metastasis, but inferior vena cava infiltraion with dissemination to the right atrium is an infrequent event.

**Presentation of case:**

This is the first case of surgical treatment of recurrent liver metastasis with the infiltration to the inferior vena cava and to the right atrium of the heart, using a cryopreserved pulmonary homograft.

**Discussion:**

The choice of a cryopreserved pulmonary homograft was preferred by the need for a radical and wide resection of tissues involved in the metastasis, as well as to potentially reduce the risk of thrombosis in the short- and long-term postoperative period.

**Conclusion:**

The use of a cryopreserved homograft in operation undergoing cardiopulmonary bypass allowed us to perform the required volume of radical resection and to replace an extended section of the inferior vena cava.

## Introduction

Surgical treatment of infiltration or thrombosis of the inferior vena cava (IVC) in malignant liver neoplasms is associated with high risks, which are counterbalanced by possible advantages in the absence of alternative therapies [1]. In the absence of surgical treatments, the median survival rate is less than 12 months, and chemotherapy has demonstrated acceptable survival rates in few reports [2, 3]. In this report, we demonstrate the surgical treatment of a patient with a liver metastasis complicated by the thrombosis of IVC and right atrium undergoing cardiopulmonary bypass (CPB).

This work has been reported in line with the SCARE 2020 criteria [4].

### Case presentation

**Fig. 1:**
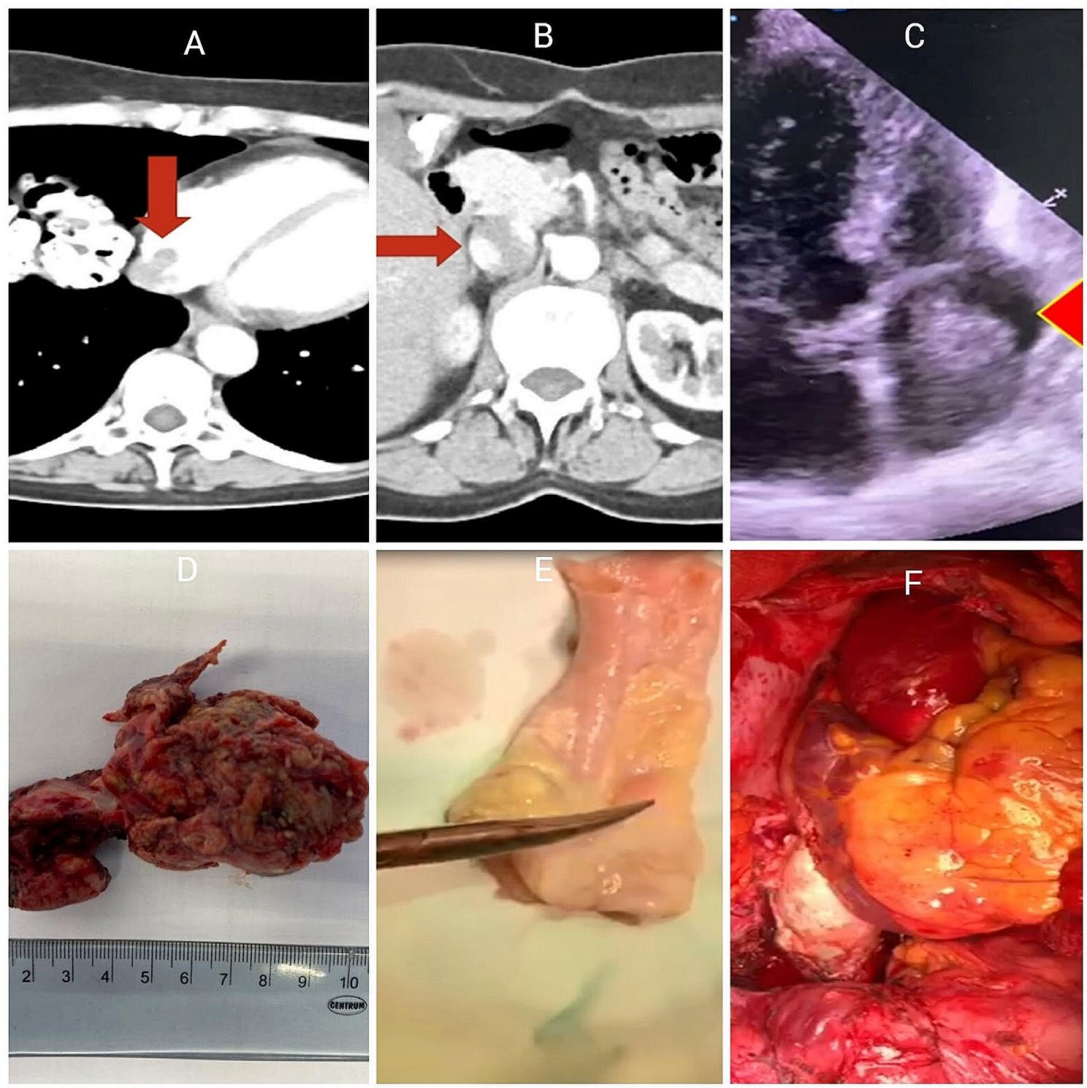
CT scan before operation, tumor trombosis into right atrium and inferior vena cava (**A**,**B**), TEE view before operation demonstrate tumor mass into right atrium (**C**), resected part of IVC and right atrium (**D**), cryopreserved pulmonary homograft 28 mm (**E**), final view (**F**)

A 60-year-old woman with a clinical picture of dyspnea at moderate physical exertion, weakness and discomfort in the heart area was admitted to the Clinic of Faculty of Surgery No. 1. In 2017, a left-sided hemicolectomy was performed for splenic flexure transverse colon cancer pT4AN2AM0 (TNM 8), histologically moderately differentiated adenocarcinoma. The patient received a total of 8 courses of adjuvant chemotherapy on the CAPOX scheme in 2017. The patient was further operated, twice for liver metastasis: in 2018, liver segments 2, 3 and 4 A were resected; in 2019, liver segmentectomy IVB was performed. After liver segmentectomy IVB patient received 8 courses of adjuvant chemotherapy on the FOLFIRI scheme in 2019 with dose reductions to avoid intolerance. We performed an echocardiography on January 2020 that showed a subtotal thrombosis of the right atrium, and other parameters were normal. Computed tomography (CT scan) revealed a hepatic segment I metastasis with inferior vena cava thrombosis, spreading into the right atrial cavity (Fig. 1 A, B).

Moving thrombotic masses in the right atrium were confirmed by transesophageal echocardiography (TEE). (Fig. 1 C).

Surgery was performed on February 5, 2020: median sternotomy and upper median laparotomy. The stage of mobilization and removal of the first segment of the liver was performed before the start of CPB. Cardiopulmonary bypass was connected by type: ascending aorta, superior vena cava and inferior vena cava (below tumor thrombosis) under normothermic conditions. After adhesiolysis, tourniquets on the superior vena cava and IVC were placed. Tourniquets are superimposed together to place venous cannulas and are squeezed so that blood does not fall below the place of standing of the cannula of the superior vena cava and above the place of standing of the cannula of the inferior vena cava. Longitudinal cavatomy was performed, the revision revealed invasion of the inferior vena cava wall up to the entry into the right atrium.

Thrombotic masses were visualized, which spread into the right atrium and were fixed to the wall, the tricuspid valve was intact. Histological examination revealed tubular adenocarcinoma of the liver and R0 edge of resection. For radical removal of the tumor conglomerate, to reduce the time of CPB and more physiologically close the major defect of the right atrium and prosthetics of the inferior vena cava, we decided to implant a pulmonary cryopreserved homograft in the position of the inferior vena cava. We performed resection of the area of the right atrium and inferior vena cava involved in the tumor process, and prosthetics of the area of inferior vena cava, right atrium with cryopreserved pulmonary homograft of 28 mm in diameter. The proximal anastomosis was formed with the widest part of the pulmonary homograph (bifurcation area), the distal anastomosis was formed with the proximal portion of the pulmonary homograft, the pulmonary valve was previously excised. The proximal and distal anastomoses were formed by a continuous winding suture using a prolene 5 − 0. The cardiopulmonary bypass time was 67 min.

Tumor, a part of the resected inferior vena cava with part of right atrium, pulmonary homograft are showed after reconstruction in (Fig. 1 D, E,F).

After surgery, we observed the patient in the intensive care unit for 24 h, and then the patient was transferred to the cardiac surgery unit, where she continued her rehabilitation. On the first day we removed postoperative drains from the thoracic cavity. On the 3rd day we removed the drainage from the abdominal cavity as well. The postoperative period was smooth and the postoperative wounds healed. However, in the postoperative period (2–3 days) due to the large wounded surface the patient began to feel dyspnea and decrease in saturation up to 90–91%. On the background of CPAP therapy for 5 days the patient’s condition showed positive dynamics, saturation 95–97%. The patient received low-molecular-weight heparin in therapeutic dosage for the first 3–4 days. Then the patient was prescribed rivaroxaban 20 mg continuously.

In postoperative period patient received 4 courses of adjuvant chemotherapy on the FOLFIRI scheme in 2020.

**Fig. 2:**
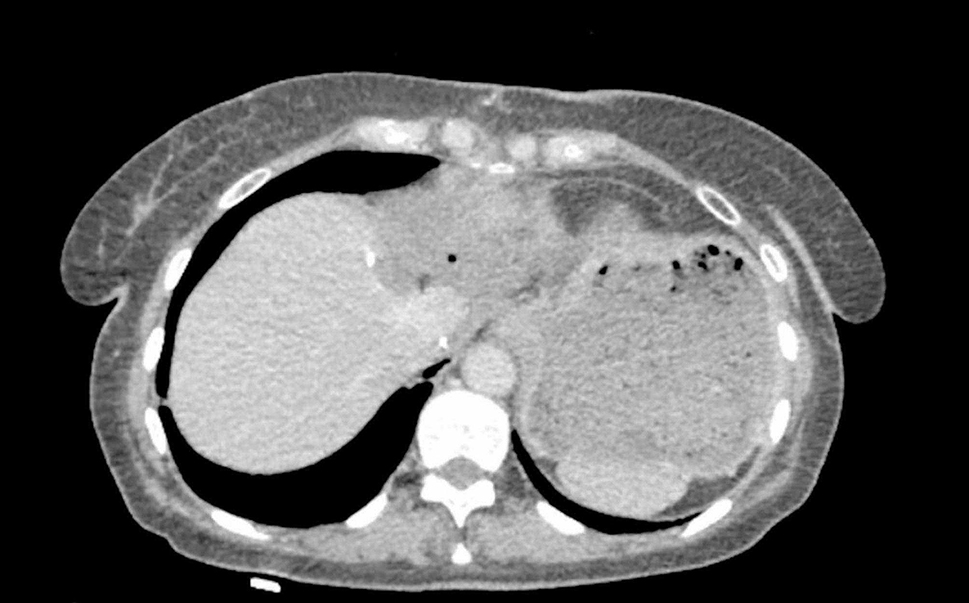
CT scan after 3 years. There is no data for recurrent

Three year after the operation according to echocardiography and CT, there was no evidence of recurrence (Fig. 2), and an adequate function of the tricuspid valve.

## Discussion

Surgical removal of thrombotic masses from the lumen of the IVC and the right atrium is the only radical treatment that can reduce the risk of systemic metastasis and sudden death due to pulmonary thromboembolism or occlusion of the tricuspid valve by a tumor thrombus [5]. We believe that with this spread of tumor thrombosis, CPB should be used for radical surgery. Normothermal parallel CPB may be sufficient, as in our case, thus, there is no need for cardioplegia and circulatory arrest. Сompression of cava veins with turnstiles as in cardiac surgery on the left heart (mitral valve surgery) provides a dry surgical field, which allows adequate revision of the right heart, tricuspid valve. As a blood-saving technology, we used coronary suction, which allowed us to return blood back to the body. To achieve hemostasis in the area of liver segment resection after the end of CPB and decanulation, we used a bipolar coagulator and a hemostatic sponge. We performed transfusion of freshly frozen plasma and cryoprecipitate. In order to replenish the coagulation factors we injecting the prothromplex. We believe that possible recurrent were also due to the fact that the patient did not tolerate previously prescribed chemotherapy scheme well, and therefore the courses were interrupting indefinitely. We consider it important to pay attention to the edge of resection during intraoperative histological examination, it should be R0, in this case we can talk about the radicality of the intervention and count on satisfactory long-term results. The correct selection of chemotherapy and immunotherapy, its tolerability also plays an important role in order to prevent the recurrence of cancer. The use of tubular hand-made prostheses made of auto or xenopericardium requires additional time for CPB and ladditional operation time, since the final volume of reconstruction was determined after the removal part of the inferior vena cava and the right atrium, it was not possible to form a neograft of the required size in advance. The use of synthetic prostheses in the position of IVC increases the risk of thrombosis and infectious complications. Thus, we preferred to use a cryopreserved pulmonary homograft of sufficient diameter in order to replace the defect of IVC and RA. According to the preoperative PET - CT, we did not detect a lesion of the lymph nodes, thus we refrained from performing lymph dissection.

We consider that the surgical method of treatment remains the method of choice. Thus, Kokudo and colleagues demonstrated that the median survival rate is higher with surgical treatment methods than with chemoembolization or chemotherapy alone [6, 7]. In another side, Chao and colleagues demonstrate a two-step approach, the first stage is thrombectomy with or without CPB, the second stage is hepatectomy. However, it is not fully known what period should be between the stages. Also, the authors do not indicate whether they stop the heart or not, apply hypothermia or not [8]. Laquaglia et al. demonstrate how with technical difficulties, an ex vivo hemihepatectomy can be performed in CPB [9]. von Riedenauer et al. described the use of veno-venous bypass for the removal of a giant liver adenoma in a 17-year-old boy [10].

## Conclusion

Surgical treatment of tumor invasion or thrombosis of the inferior vena cava and right atrium undergoing CPB seems to be a promising approach to decrease the haemorrhage and a radical intervention. Cryopreserved homograft in the position of the inferior vena cava does not affect on the hemodynamics of the right parts of the heart. The use of cryopreserved homografts in the position of the inferior vena cava is an acceptable alternative to auto or xenopericardial conduit as well as synthetic prostheses. However a longer observation period is needed to form final conclusions.

## Data Availability

Not applicable.
